# The patterns of loss of correction after posterior wedge osteotomy in ankylosing spondylitis-related thoracolumbar kyphosis: a minimum of five-year follow-up

**DOI:** 10.1186/s12891-017-1834-4

**Published:** 2017-11-17

**Authors:** Mu Qiao, Bang-ping Qian, Sai-hu Mao, Yong Qiu, Bin Wang

**Affiliations:** 1 Department of Spine Surgery, Drum Tower Hospital of Nanjing University Medical School, Zhongshan Rd 321, Nanjing, 210008 China; 2 Medical School of Southeast University, Nanjing, China

**Keywords:** Ankylosing spondylitis, Thoracolumbar kyphosis, Middle-term, Pedicle subtraction osteotomy, Surgical outcome, Ossification, Pattern, Loss of correction, Non-instrumented segment

## Abstract

**Background:**

Short-term studies have demonstrated good surgical outcomes after pedicle subtraction osteotomy (PSO) in ankylosing spondylitis (AS) patients, but there is a paucity of literature focused on middle-term results, especially regarding patterns of loss of correction. The objective of this study is to assess the durability of surgical outcomes and the patterns of loss of correction in thoracolumbar kyphosis secondary to AS following lumbar PSO with over 5-year follow-up.

**Methods:**

We performed a retrospective review of 155 consecutive AS patients undergoing lumbar PSO from January 2001 to December 2011. Twenty-four patients were included with an average follow-up of 6.9 years (range, 5–15 years). Radiographical evaluations included global kyphosis (GK), lumbar lordosis (LL), sagittal vertical axis, spinal-sacral angle, kyphosis of proximal non-fused segments (KPNS), angle of fused segments (AFS), osteotomized vertebra angle (OVA), distal intervertebral disc wedging (DIDW) and proximal junctional angle. Meanwhile, clinical outcomes were assessed by the Oswestry disability index (ODI) and Numerical rating scale (NRS).

**Results:**

The average correction per PSO segment was 34.9°. Significant improvement in sagittal parameters were found postoperatively, and no obvious deterioration was noticed during the follow-up. Mild loss of correction in GK (2.82°) and LL (3.77°) were observed at the final follow-up (*P* < 0.05). The KPNS and DIDW increased from 26° and −5.0° postoperatively to 30° and −2.2° at the final follow-up (*P* < 0.05), respectively. In contrast, no significant diminishment was identified in OVA and AFS (*P* > 0.05). The ODI and NRS improved significantly from 20.6 and 6.6 preoperatively to 5.9 and 2.3 at the final follow-up (*P* < 0.05).

**Conclusions:**

PSO is an effective procedure for treating AS-related thoracolumbar kyphosis and can maintain sustained surgical outcomes during the middle-term follow-up. The loss of correction was mainly attributable to non-instrumented segments without fully ossified bridging syndesmophyte in the thoracolumbar region instead of instrumented levels.

## Background

Ankylosing spondylitis (AS) is a chronic rheumatic disease characterized by axial skeleton and peripheral joints inflammation [1]. The bony ankyloses of the entire spine eventually produced a fixed thoracolumbar kyphosis leading to sagittal imbalance and restricted psychosocial activities [2]. Thus, surgical intervention is usually indicated to restore sagittal alignment and to reduce functional impairments.

Dramatic improvements from pedicle subtraction osteotomy (PSO) in sagittal alignment for thoracolumbar kyphosis has been well documented with short-term follow-up [2–6]. However, loss of correction could not be ignored in the follow-up period. In a recent study, Qian et al. [3] demonstrated loss of correction of 3.6° and 2.5° in global kyphosis (GK) and LL after posterior wedge osteotomy with a mean follow-up of 3 years. Besides, Kim et al. [7] reported loss of 4.8° in GK correction for AS-related kyphosis over 2 years postoperatively. In the presence of existing thoracic kyphosis, the loss of correction may place the fusion zone under tension due to the long lever arm, thereby resulting in the implant failure and progressive loss of correction [8–11].

Considering the above-mentioned potential risks caused by loss of correction, an enhanced understanding of surgical outcomes in PSO-treated AS patients with longer follow-up is needed. Furthermore, as the pattern related to loss of correction after lumbar PSO surgery remains unclear, we sought to evaluate the surgical outcomes in a medium to long-term follow-up period, with particular attention to the magnitude and location of loss of correction.

## Methods

### Population

**Fig. 1:**
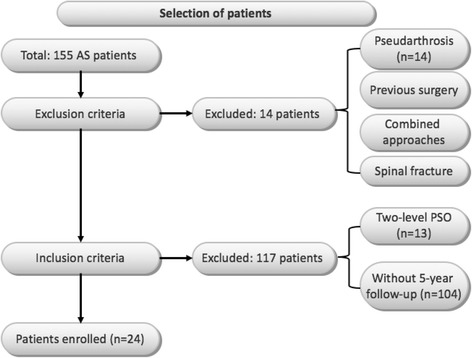
The flow diagram shows a clear process of the selection of patients

This study was approved by the Medical Ethics Committee of Medical School of Nanjing University (the ethics approval number provided by the board was 2,011,052). Written informed consent was obtained from all patients prior to testing. The medical records of 155 consecutive AS patients who had undergone lumbar PSO for fixed sagittal imbalance between January 2001 and December 2011 were reviewed. The inclusion criteria listed as follows: (1) underwent single-level PSO; (2) with a minimum of five-year follow-up. The exclusion criteria were: (1) spinal fracture; (2) pseudoarthrosis; (3) previous spinal surgery; and (4) combined anterior-posterior procedure. Based on the above-mentioned inclusion and exclusion criteria, 9 patients were excluded due to undergoing double-level-PSO while 12 patients who developed pseudarthrosis preoperatively were also removed. 79 patients were excluded due to lack of over 2-year follow-up data. Moreover, 31 patients with more than 2-year follow-up but without over 5-year follow-up data were excluded as well (Fig. 1). Finally, a total of twenty-four AS patients consisting of 20 males and 4 females with a mean age of 38 years (range, 23–56 years) were included in this study eventually. On average, the follow-up period was 6.9 years (range, 5–15 years). Totally, PSO was performed at L1 in 7 cases, at L2 in 14 cases and at L3 in 3 cases. Among the 24 patients, three types of rods were used: (1) 6.35 mm stainless rod in 7 patients; (2) 5.5 mm titanium rod in 9 patients and (3) 6.35 mm titanium rod in 8 patients. The details regarding the use of bone grafts were described as above-mentioned. Neither iliac bone nor artificial bone was used for fusion. Regarding postoperative external fixation, all patients were allowed to mobilize with a thoracolumbosacral orthosis for 3 months.

### Radiographic evaluation and clinical assessment

**Fig. 2:**
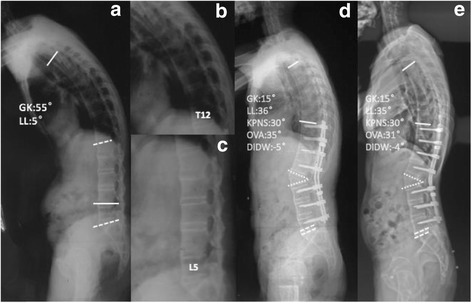
Forty seven-year-old male (**a**) with fully ossified anterior longitudinal ligament (**b**)(**c**) underwent L2 PSO (**d**). No loss of correction in GK and LL was identified at the follow-up of 72 months (**e**). (OVA: osteotomized vertebra angle; KPNS: kyphotic angle of proximal non-fused segments; DIDW: distal intervertebral disc wedging; GK: global kyphosis; LL: lumbar lordosis)

**Fig. 3:**
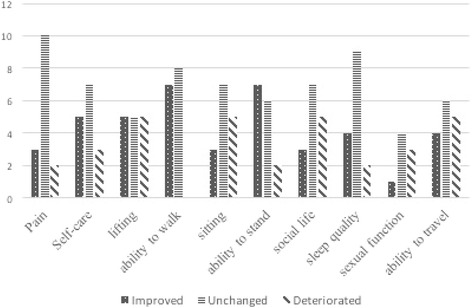
The diagram unfolds a clear comparison between preoperative and the final follow up results of all the items in ODI as a whole in three aspects: improved, unchanged and deteriorated (*n* = 15). ODI indicates oswestry disability index

Radiological assessment was performed preoperatively, postoperatively, and at the final follow-up. The sagittal parameters included: (1) global kyphosis (GK) [3]: the angle between the superior endplate of the maximally tilted upper end vertebra and the inferior endplate of the maximally tilted lower end vertebra on the full-length lateral radiographs (Fig. 2a); (2) lumbar lordosis (LL) [11]: the angle between the superior L1 endplate and the S1 endplate; The angle is positive if the curve is lordotic and negative if the curve is kyphotic (Fig. 2a); (3) sagittal vertical axis (SVA) [12]: the distance between the C7 plumb line and the posterior-superior corner of S1; (4) spinal-sacral angle (SSA) [13]: the angle between the line connecting the centroid of C7 vertebral body and the midpoint of the sacral endplate; (5) kyphotic angle of proximal non-fused segment involved in the GK (KPNS): the angle between the superior endplate of the preoperative maximally tilted upper end vertebra and the superior endplate of upper end vertebra (Fig. 2d); (6) angle of fused segments (AFS); (7) proximal junctional angle (PJA) [14]: determined by the angle between the caudal endplate of the upper instrumented vertebra and the cranial end plate of two vertebrae above. Proximal junctional kyphosis (PJK) is defined as a kyphosis exceeding 10° and an increase of the PJA of at least 10° compared to the preoperative value; [6] (8) osteotomized vertebra angle (OVA): the angle between superior and inferior endplate of osteotomized vertebra (Fig. 2d); and (9) the sum of distal non-fused intervertebral disc wedging (DIDW) (Fig. 2d). The angle is positive if the curve is lordotic and negative if the curve is kyphotic in LL. Meanwhile, the PJA, KPNS and DIDW were only obtained from postoperative and follow-up radiographs (Fig. 2d and e). Each parameter was measured by an orthopedic resident for 3 times using surgimap software (Nemaris, New York, NY) and the average values were calculated [15].

The radiographic changes of bilateral hips were evaluated with the BASRI-hip scoring system preoperatively and at the final follow-up. The system was categorized into five severity grades based on the discriminating features (0 = no disease, 1 = suspicious disease, 2 = minimal disease, 3 = moderate disease, and 4 = severe disease) [16].

All patients were functionally examined with the Oswestry Disability Index (ODI) scores and Numerical rating scale (NRS) preoperatively and at the last follow-up. The ODI scores consisted of ten items: pain, lifting, ability to care for oneself, ability to walk, ability to sit, sexual function, ability to stand, social life, sleep quality, and ability to travel. Each item is followed by six statements describing different potential scenarios in the patient’s life. Meanwhile, each question is scored on a scale of 0–5 (0 = the least amount of disability, 5 = most severe disability) [17].

### Statistical analysis

Statistical analysis was done using SPSS software (version 12.0; 2003; SPSS, Inc. Chicago, IL, USA). Analysis of variance was used to compare the preoperative, postoperative and the final follow-up radiological and clinical data with paired t-test. A *p*-value less than 0.05 was considered as statistically significant.

## Results

### Radiological outcomes

GK, LL, SVA and SSA were significantly improved from baseline to the final follow-up. GK and LL changed to 29.43 ± 11.81° and 43.86 ± 9.37° with a mean correction loss of 2.82° and 3.77° at the latest follow-up, respectively (*P* < 0.05). With respect to SVA and SSA, no significant deterioration was found from five-year postoperative radiographic data (*P* > 0.05) (Table 1). KPNS and DIDW increased from 26° and −5.0° postoperatively to 30° and −2.2° at the last follow-up (*P* < 0.05). Conversely, no significant difference was noticed in OVA and AFS (*P* > 0.05). At the final follow-up, eleven AS patients noted deteriorated results in the items of ODI such as sitting, social life and travel. Among these patients, the increased BASRI-hips scores was identified in nine (Table 2) [15].

**Table 1:** Radiographic and clinical assessment of preoperative, postoperative and the final follow-up data (*n* = 24)

Parameters	Pre-op	Post-op	*P*	Follow-up	*P*	Loss of correction
GK (°)	69.71 ± 16.61	26.62 ± 12.27^a^	<0.001	29.43 ± 11.81^b^	<0.05	2.82 ± 2.42
LL (°)	8.95 ± 12.55	47.64 ± 10.01^a^	<0.001	43.86 ± 9.37^b^	<0.05	3.77 ± 2.94
SVA (mm)	154.51 ± 52.94	34.95 ± 45.42^a^	<0.001	51.85 ± 44.03	0.175	–
SSA (°)	79.11 ± 11.53	108.68 ± 7.73^a^	<0.001	107.79 ± 10.35	0.581	–
OVA (°)	–	−29.17 ± 7.71	–	−28.04 ± 7.90	0.057	–
KPNS (°)	–	25.95 ± 12.35	–	29.76 ± 13.55^b^	<0.05	–
DIDW (°)	–	−5.0 ± 8.60	–	−2.23 ± 7.46^b^	<0.05	–
AFS (°)	–	22.76 ± 7.58	–	22.94 ± 7.74	0.083	–
PJA (°)	–	9.70 ± 5.25	–	12.30 ± 5.11^b^	<0.05	–
ODI	20.57 ± 13.63	–	–	6.63 ± 6.44^a^	<0.05	
NRS	5.94 ± 3.28	–	–	2.31 ± 2.24^a^	<0.05	

*GK* global kyphosis, *LL* lumbar lordosis, *SSA* spinal-sacral angle, *SVA* sagittal vertical axis, *OVA* osteotomized vertebra angle, *KPNS* kyphosis of non-fused segment, *DIDW* distal intervertebral disc wedging, *AFS* angle of fused segments, *PJA* proximal junctional angle, *ODI* Oswestry Disability Index, *NRS*: Numerical Rating Scale; “^a^”: statistically significant in comparison with the preoperative result; “^b^”: statistically significant when compared to the postoperative result

**Table 2:** The BASRI-hips of patients with deteriorated items in ODI (*n* = 11)

Patient	BASRI-hips				Oswestry disability index						
	Left side		Right side		Pain	Self-care	Lifting	Sitting	Sleeping	Social life	Travel
	Pre-op	Fw	Pre-op	Pre-op							
1	0	1	0	1	+	+					
2	4	4	2	4			+	+			+
3	1	1	1	1		+					
4	1	3	2	3	+	+	+	+			
5	1	2	1	2					+	+	+
6	0	0	2	1			+		+	+	+
7	1	1	2	2						+	+
8	1	2	2	2				+		+	+
9	1	1	1	1			+				
10	1	1	2	3				+		+	
11	1	2	1	2				+			

*Fw* the final follow-up; **“+”**: deteriorated at the final follow-up in comparision with preoperative data

### Clinical outcomes

Complete clinical data was available in fifteen patients (62.5%). The ODI and NRS improved significantly from 20.57 and 6.63 preoperatively to 5.94 and 2.31 at the final follow-up (*P* < 0.05). Most of patients showed functional improvement and pain relief at the final follow-up. Notably, five patients demonstrated deterioration in social life and travel item at the last follow-up. Meanwhile, the diminishment in the ability of sitting and lifting was identified in five and four patients when compared to the preoperative results, respectively. Sleeping disorder was noticed in two patients and the disability to care for themselves was observed in five patients (Fig. 3) [15].

### Comorbidities

Chin-on chest deformity was observed in one case at the follow-up of 72 months. Subsequently, PSO at C7 segment was performed to restore the horizontal gaze. Cervical fracture after minor trauma occurred in one patient at the time of fifteen years postoperatively and underwent the anterior cervical discectomy and fusion.

### Complications

Late deep infection was observed in only one case at the final follow-up of 84 months. Wound was regularly cleaned and healed uneventfully. No patients developed with pseudarthrosis at the site of the osteotomy. The measures taken to prevent pseudarthrosis consisted of: (1) the decancellation bone from the osteotomized vertebral body and the cortical bone chips from spinous process were placed tightly on the surfaces of laminae posteriorly and posterolaterally; (2) the decancellation of bone was uniform throughout the vertebral body to produce a symmetric closure and to achieve complete bone apposition centrally and laterally at the osteotomy site; and (3) after ensuring that there is no compression of dural sac and nerve root, the width of osteotomized gap of the posterior elements should be as narrow as possible to avoid a wide defect between two adjacent laminae. Also, no PJK and instrumentation failures was detected at the follow-up.

## Discussion

A fixed and rigid thoracolumbar kyphosis caused by AS often leads to sagittal imbalance, a downward tilt of head and muscle fatigue [18, 19]. Improvement from lumbar PSO in spinopelvic alignment has been well documented [18–20]. The results of our study were in line with the findings of previous studies [3, 18, 21], revealing that satisfactory surgical correction could be achieved in AS patients following one-level PSO. The mean correction per PSO level was 34.9° and was comparable to published literature (34.5°) [20]. And all radiographic parameters improved significantly after surgery without obvious deterioration at the final follow-up. No significant difference was noted in terms of OVA (1.13°), which indicated the rapid consolidation at the osteotomy site [22, 23]. Meanwhile, instrumentation-related complications at the final visit was not identified, such as PJK, hardware failure and pseudoarthrosis. The lower rate of long-term complication may also be attributed to the substantial osseous union at the osteotomy site and stable biomechanically environment after correction surgery.

However, loss of correction has also been reported by several authors in the follow-up period. Qian et al. [12] assessed short-term radiological outcomes in thirty-six PSO-treated AS patients and found a worsening of GK (3.1°) combined with decreased LL (2.6°) two years after surgery. In a study of 51 patients, Chang et al. [24] evaluated radiographic changes and identified similar increases in thoracic kyphosis and deteriorated LL following posterior wedge osteotomy at the last visit of 3.6 years postoperatively [18]. Though widely reported in the short-term studies, the magnitude and location of loss of correction remains unknown over five years postoperatively. Accordingly, a better understanding of long-term surgical outcomes after PSO is necessarily warranted, particularly with regard to the pattern of loss of correction.

**Fig. 4:**
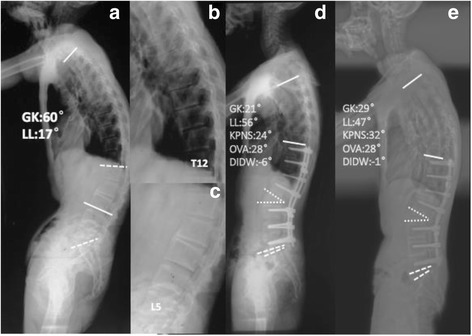
29-year-old male (**a**) without fully ossified thoracolumbar structure (**b**)(**c**) underwent L2 PSO (**d**). Moderate loss of correction in GK and lumbar lordosis LL at the follow-up of 84 months were identified (**e**). The loss of correction mainly resulted from the increased KPNS and DIDW. (OVA: osteotomized vertebra angle; KPNS: kyphotic angle of proximal non-fused segments; DIDW: distal intervertebral disc wedging; GK: global kyphosis; LL: lumbar lordosis)

Mild loss of correction in GK (2.82°) and LL (3.77°) were identified from the final follow-up radiographs when compared with postoperative data in our study. With regard to AFS, no statistically significant difference was noted between immediate postoperative and the last follow-up. The results of our study revealed that solid fusion was achieved and loss of correction would be less likely to occur in the operated segments, which was in line with a previous study by Chen et al. [25]. In contrast to AFS, there were significant changes in KPNS and DIDW during follow-up period. Therefore, the loss of correction was mainly ascribed to non-fused segments. In this series, two mechanisms leading to loss of correction were identified. Initially, the increased KPNS and DIDW were mostly associated with the deformation of proximal and distal structure, respectively. Theoretically, the disc status would not be easily altered when solid anterior column was provided by fully ossified bridging syndesmophytes. While for AS patients without fully ossified anterior longitudinal ligament, proximal and distal discs is prone to developing collapse under the active inflammation. In the present study, deterioration more than 2° in KPNS and DIDW was found in fourteen patients without fully ossified structure, which supported the above-mentioned hypothesis (Fig. 4). Secondly, the mobility of non-fused segments adjacent to the operated levels may lead to the change of proximal or distal non-instrumented segments, resulting in the loss of correction. Thus, it should be emphasized that examination of ESR and CRP level and application of anti-inflammation medication should be regularly performed for AS patients without fully ossified bridging syndesmophytes during the follow-up period to prevent the loss of correction [23, 24]. Nevertheless, the above-mentioned results were not completely in accordance with the conclusions reported by several previous studies in adult spinal deformity (ASD) [9–11]. In a study of 183 ASD patients reported by Theologis et al. [11], the instrumented lumbar levels were assumed to be the primary cause for loss of lumbar sagittal correction in long adult deformity constructs.

It has been well accepted that spinal alignment was related to quality of life [26, 27]. In agreement with previous literatures, [18–20, 24–28] early pain relief, improved cosmesis and function from a rigid fixation and sustained spinal realignment resulted in the better ODI and NRS scores in our series. However, deterioration was noted in certain items of ODI at the final follow-up regardless of the maintenance of radiological parameters, such as pain, self-care ability, social life, sleep quality, ability to travel and ability to sit. In terms of the domain of pain and sitting, an awareness of the potential factors contributing to the diminishment was the hip involvement by persistent inflammation. In the current study, the increased BASRI-hips scores at the final follow-up was identified in all the five patients with deteriorated results in the item of sitting. In addition, four patients with worse BASRI-hips scores were found to have deteriorated outcome in the item of social life and travel. One possible explanation is that persistent pain and disability of sitting resulting from the hips may lead to the incapacity of driving or riding. Overall, it is worth noting that underlying inflammation involving the hips thereby leads to the decreased function at the long-term follow-up. Furthermore, in terms of postoperative lifestyle guidance, generally, heavy lifting (over 10 kg) should be avoided within 9 months after surgery. Meanwhile, farm work or heavy labor need to be determined on an individualized basis. Additionally, patients should be encouraged to abstain from alcohol and cigarette to prevent non-union, implant failure and to improve the clinical outcomes.

Although the durable surgical outcomes following lumbar PSO in AS-related kyphosis patients with long follow-up period are revealed in this study, we noticed that one limitation of the present study was retrospective in design and the sample size is relatively small. Another limitation is the lack of pelvic parameters to assess whether the spinopelvic harmony is maintained. Future study examining the long-term radiological and clinical outcomes with more parameters is certainly warranted.

## Conclusion

Durable and sustained surgical outcomes in this series showed that lumbar PSO is an effective and safe treatment for thoracolumbar kyphosis secondary to AS in the middle-term follow-up. The detected loss of correction was mainly in virtue of non-instrumented levels without fully ossification in the thoracolumbar region instead of instrumented segments as reported in most of degenerative spinal deformity.

Deterioration in certain domains of ODI may be ascribed to the hip involvement with persistent inflammation. For postoperative care, regular evaluation of underlying inflammation is beneficial for the maintenance of kyphosis correction and avoidance of hip joint dysfunction.

## Abbreviations

AFS: angle of fused segments
AS: Ankylosing spondylitis
DIDW: the sum of distal non-fused intervertebral disc wedging
GK: global kyphosis
KPNS: kyphotic angle of proximal non-fused segment involved in the global kyphosis
LL: lumbar lordosis
NRS: Numerical rating scale
ODI: Oswestry Disability Index
OVA: osteotomized vertebra angle
PJA: proximal junctional angle
PJK: proximal junctional kyphosis
PSO: Pedicle subtraction osteotomy
SSA: spinal-sacral angle
SVA: sagittal vertical axis
